# Hybrid quantum systems with high-T$_c$ superconducting resonators

**DOI:** 10.1038/s41598-023-41472-z

**Published:** 2023-09-01

**Authors:** Z. Velluire-Pellat, E. Maréchal, N. Moulonguet, G. Saïz, G. C. Ménard, S. Kozlov, F. Couëdo, P. Amari, C. Medous, J. Paris, R. Hostein, J. Lesueur, C. Feuillet-Palma, N. Bergeal

**Affiliations:** 1 Laboratoire de Physique et d’Étude des Matériaux, ESPCI Paris, Université PSL, CNRS, Sorbonne Université, Paris, France; 2 Laboratoire National de Métrologie et d’Essais (LNE), 29 Avenue Roger Hennequin, 78197 Trappes, France; 3 CNRS, Institut Fourier, Université Grenoble Alpes, 38000 Grenoble, France; 4 Université Grenoble Alpes, INRIA, 38000 Grenoble, France; 5 My Cryo Firm, 20 Villa des Carrières, 94120 Fontenay-sous-Bois, France

**Keywords:** Superconducting devices, Condensed-matter physics

## Abstract

Superconducting microwave resonators are crucial elements of microwave circuits, offering a wide range of potential applications in modern science and technology. While conventional low-T$_c$ superconductors are mainly employed, high-T$_c$ cuprates could offer enhanced temperature and magnetic field operating ranges. Here, we report the realization of $\textrm{YBa}_2\textrm{Cu}_3\textrm{O}_{7-\delta }$ superconducting coplanar waveguide resonators, and demonstrate a continuous evolution from a lossy undercoupled regime, to a lossless overcoupled regime by adjusting the device geometry, in good agreement with circuit model theory. A high-quality factor resonator was then used to perform electron spin resonance measurements on a molecular spin ensemble across a temperature range spanning two decades. We observe spin-cavity hybridization indicating coherent coupling between the microwave field and the spins in a highly cooperative regime. The temperature dependence of the Rabi splitting and the spin relaxation time point toward an antiferromagnetic coupling of the spins below 2 K. Our findings indicate that high-Tc superconducting resonators hold great promise for the development of functional circuits. Additionally, they suggest novel approaches for achieving hybrid quantum systems based on high-T$_c$ superconductors and for conducting electron spin resonance measurements over a wide range of magnetic fields and temperatures.

## Introduction

Resonators are essential building blocks of microwave circuits in which they can perform a wide variety of functions, ranging from narrow-band filtering to enhancing the interaction between electromagnetic waves and quantum systems. In practice, the performance of a resonator, such as its ability to store energy, is often limited by material losses. Using superconducting materials as the conductive part significantly reduces dissipation, which in turn leads to a large increase in the internal quality factor of the resonator. This also gives flexibility in the design, allowing for both compact lumped-element or distributed form implementations^1^. In addition, the resonance frequency can be tuned by incorporating Josephson junctions and SQUIDs into the resonator acting as temperature or magnetic field tunable inductive elements^2,3^. Thanks to their versatility, superconducting microwave resonators have attracted extensive research interest, with potential applications in fields such as wave detection^4,5^, parametric amplification^6,7^ and quantum information processing^8^. In particular, major progress has been achieved in the coupling of microwave cavity modes to quantum objects in hybrid systems, including superconducting^9,10^ or spin qubits^11,12^, paramagnetic spin ensemble in molecules^13–16^ and crystals^17,18^, magnons in magnetic materials^19,20^, nanomechanical resonators^21,22^ and cold atoms^23,24^.

So far, most resonators have been made with conventional low-T$_c$ superconducting materials such as Al, Nb and NbN through well-established fabrication processes. Their properties have been well characterized and reported in several articles^1,25^. In this context, high-T$_c$ (HT$_c$) superconducting materials, such as cuprates, offer several advantages. Although, they can not compete with their low-T$_c$ counterparts for ultra-high quality factors due to intrinsic microwave dissipation, they still offer sufficient performance for many applications and can operate at higher temperatures and in stronger magnetic fields. In this letter, we report on a detailed study of HT$_c$ superconducting coplanar waveguide (CPW) resonators made in $\textrm{YBa}_2\textrm{Cu}_3\textrm{O}_{7-\delta }$ thin films. We show that by decreasing the capacitive coupling with the external circuit, a continuous transition occurs from an overcoupled regime, in which the quality factor of the resonator is determined by the coupling, to an undercoupled regime in which the quality factor is limited by the losses of the superconductor. The experimental results, including resonance frequency, quality factor, and insertion loss, are in good agreement with the predictions of circuit model theory. Finally, to assess the application potential of such resonators, we conducted electron spin resonance (ESR) experiments on a 2,2-diphenyl-1-(2,4,6-trinitrophenyl) hydrazyl (DPPH) molecular spin ensemble from 0.1 to 17 K. We observe an avoided crossing characteristic of spin-cavity hybridization in a highly cooperative regime, which reveals a transition to an antiferromagnetic state below 2 K. 

## Design and fabrication of the resonators

**Figure 1:**
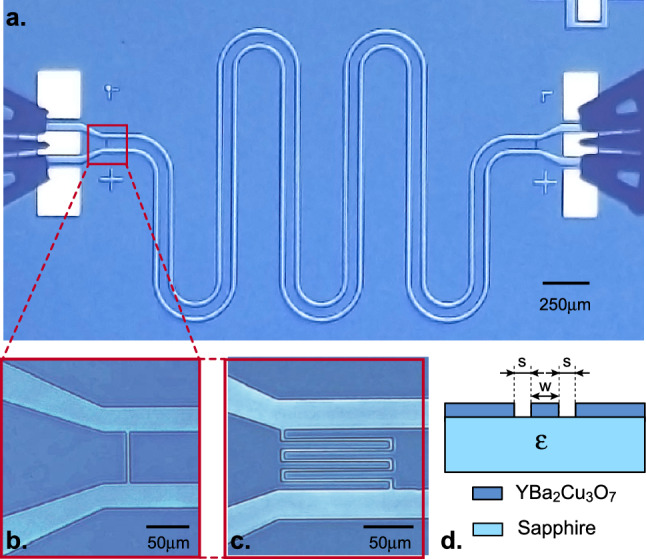
$\textrm{YBa}_2\textrm{Cu}_3\textrm{O}_{7-\delta }$ CPW resonators. (**a**) Optical picture of resonator R4 with microwave probes on the contact pads. (**b**) Picture of a 1$\mu \textrm{m}$ gap input capacitor (R4). (**c**) Picture of a 3+3 fingers gap input capacitor (R2) (**d**) Scheme of the vertical cross-section of the resonators showing the central conductor of width *w* and the gap with the lateral grounds *s*.

In this work, we fabricated $\lambda /2$ coplanar waveguide resonators in 200 nm thick commercial $\textrm{YBa}_2\textrm{Cu}_3\textrm{O}_{7-\delta }$ thin films from Ceraco ($T_c$ $\simeq$ 88 K), grown on a sapphire substrate with a CeO${_2}$ buffer layer, and covered with a 20 nm thick Au protective layer. Unlike discrete element LC resonators, such resonators consist of a finite segment of a transmission line having some distributed inductance and capacitance to ground. Resonators were defined by Ar ion beam etching through an AZ5214 optical resist patterned by laser lithography. Alternatively, selective amorphization of the $\textrm{YBa}_2\textrm{Cu}_3\textrm{O}_{7-\delta }$ layer by high-energy ion implantation could advantageously replace the etching step^26,27^. Finally, additional Ti/Au contact pads were deposited by lift-off before removing the protective gold layer. The resonators consist of a *w*= 60 $\mu \textrm{m}$ wide central conductor, separated from two lateral ground planes by a gap *s* = 30 $\mu \textrm{m}$. To avoid crosstalk between resonators having similar resonance frequencies on the same chip, the resonator length was varied from 10.9 to 11.5 mm (Fig. 1a and Table 1).

The central conductor input and output are symmetrically coupled to the external circuit through either gaps (Fig. 1b) or interdigitated finger (Fig. 1c) capacitors, enabling the exploration of a wide range of coupling. A total of eight different types of resonators, labeled R1 to R8, are considered in this work (Fig. 1 and Table 1). Because input and output capacitors impose a current node, resonances occur at frequencies $f_n$ = $\frac{\omega _n}{2\pi }$ = $n\frac{c}{2l\sqrt{\epsilon _{\textrm{eff}}}}$, where *n* = 1, 2, 3... is the order of the resonance mode, *c* is the velocity of light in vacuum and $\epsilon _{\textrm{eff}}$ $\simeq$ $\frac{\epsilon _r+1}{2}$ $\simeq$ 5.5 is the relative effective permittivity ($\epsilon _r$ is the relative permittivity of sapphire)^28^. The characteristic impedance of the CPW resonator is $Z_0$ $\approx$ $\sqrt{L_l/C_l}$ $\approx$ 52.02 $\Omega$ where $L_l$ $\approx$ 4.14 $\times$ 10$^{-7}$ H.m$^{-1}$ and $C_l$ $\approx$ 1.48 $\times$ 10$^{-10}$ F.m$^{-1}$ are the resonator geometrical inductance and capacitance per unit length, respectively, that can be calculated using conformal mapping^29^. The influence of the kinetic inductance can be neglected compared to the geometrical one ($L_l$) at temperature much lower than $T_c$.

## Results and discussion

The properties of the CPW resonators were measured using a low vibration cryogen-free microwave probe station from MyCryofirm having a base temperature of 4 K. The system is equipped with a long focal length optical microscope and piezoelectric motors to position microwave Z-probes [0–50GHz] (FormFactor) on the sample of interest with a precision of 5 $\mu$m (Fig. 1a). In this experiment, the probes were connected to a 20 GHz Vector Network Analyzer (ZNB Rohde &Schwartz) through a set of cryogenic and room temperature coaxial cables. Prior to the measurements, a full two-port calibration of the microwave set-up was performed using a cryogenic calibration kit located next to the sample. Such a procedure is necessary to determine the scattering matrix parameters $S_{ij}$ of the resonators. In all our measurements, the power applied to the resonators was kept sufficiently low to avoid any non-linear effect^30^, with the exception of the high power measurement reported in Fig. 6.

**Table 1:** Description of the input–output capacitors type for each of the resonators

name	coupling + length	name	coupling + length
R1	6 + 6 fing. C$_c$ = 92 fF l = 11.52 mm	R5	gap 10$\mu \textrm{m}$ C$_c$ = 4.81 fF l = 10.92 mm
R2	3 + 3 fing. C$_c$ = 39 fF l = 11.19 mm	R6	gap 30$\mu \textrm{m}$ C$_c$ = 2.74 fF l = 11.19 mm
R3	1 + 1 fing. C$_c$ = 17 fF l = 11.13 mm	R7	gap 60$\mu \textrm{m}$ C$_c$ = 1.54 fF l = 11.19 mm
R4	gap 1$\mu \textrm{m}$ C$_c$ = 9.91 fF l = 10.92 mm	R8	gap 100$\mu \textrm{m}$ C$_c$ = 0.81 fF l = 11.42 mm

The values of $C_c$ are obtained by finite elements method based electromagnetic simulations.

**Figure 2:**
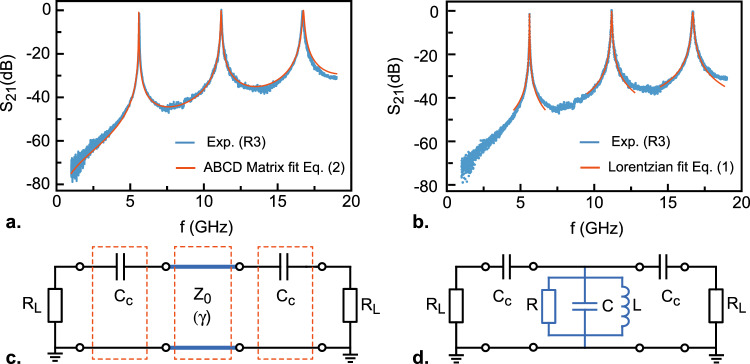
Comparison between the broadband ABCD matrix approach and the Lorentzian approximation. (**a**) Magnitude of $S_{21}$ measured at T = 8 K on resonator R3 showing three resonances at frequencies $f_1$ $\simeq$ 5.61 GHz, $f_2$ $\simeq$ 11.18 GHz and $f_3$ $\simeq$ 16.68 GHz. Data are fitted using the matrix model (Eq. (2)). (**b**) Same experimental data than in panel a showing Lorentzian fits for each resonance (Eq. 4), giving quality factors $Q_{\textrm{L}}$ = 416 for $f_1$, $Q_{\textrm{L}}$ = 257 for $f_2$ and $Q_{\textrm{L}}$ = 230 for $f_3$. (**c**) Equivalent electrical circuit used to compute the transmission $S_{21}$ with the ABCD matrices model. It includes input-output coupling capacitances $C_c$ and a $\lambda /2$ segment of CPW transmission line of impedance $Z_0$ and wave propagation coefficient $\gamma$. Each element in the red dashed line boxes is described by a single matrix (Eq. 1). $R_L$ = 50$\Omega$ denotes the impedance of the VNA. In this model, we neglect the capacitance to ground at the input and output of the resonator. (**d**) Equivalent RLC electrical circuit in the Lorentzian approximation.

Figure 2a shows the magnitude of the transmission in dB for the resonator R3 measured on the full frequency range [0.1–20 GHz]. Three resonance peaks are visible, corresponding to the fundamental mode *n* =1 and the two first harmonic modes *n* = 2 and *n* = 3, respectively. To analyze these data, we first consider the scheme shown in Fig. 2c where the resonator is represented by a transmission line segment of length *l* coupled to the external circuit through a capacitance $C_c$ on both sides. Each section is modeled by a transmission (ABCD) matrix that relates the voltages and currents at their two ports. ABCD coefficients for standard circuit elements can be found in microwave engineering textbooks^28^. The total matrix of the three cascaded networks in series is obtained by a simple product:

1 $$\begin{aligned} \begin{pmatrix} A &{} \quad B\\ C &{} \quad D\\ \end{pmatrix} = \begin{pmatrix} 1 &{} \quad \frac{1}{iC_c\omega }\\ 0 &{} \quad 1 \end{pmatrix} \begin{pmatrix} \cosh (\gamma l) &{} \quad Z_0\sinh (\gamma l)\\ \frac{1}{Z_0}\sinh (\gamma l) &{} \quad \cosh (\gamma l) \end{pmatrix} \begin{pmatrix} 1 &{} \quad \frac{1}{iC_c\omega }\\ 0 &{} \quad 1 \end{pmatrix} \end{aligned}$$

where $\gamma =\alpha +i\beta$ is the transmission line complex wave propagation coefficient ($\alpha$ is the attenuation constant and $\beta$=$\frac{\omega \epsilon _{\textrm{eff}}}{c}$ is the wave number). Finally, the scattering transmission coefficient $S_{21}$ of the resonator is expressed as a function of the abcd parameters^28^

2 $$\begin{aligned} S_{21}=\frac{2}{A+B/R_{\textrm{L}}+CR_{\textrm{L}}+D} \end{aligned}$$

where $R_L$ is the real part of the load impedance. The experimental transmission spectrum in Fig. 2a can be fitted with Eqs. (1) and (2) leading to $C_c$ = 22 fF, $\alpha$ = 1.5 $\times$10$^{-2}$ m$^{-1}$ and $\epsilon _{\textrm{eff}}$ = 5.5. The latter corresponds to $\epsilon _r$ $\simeq$ 10 for the sapphire substrate. A very good agreement is obtained throughout the entire frequency range, which validates both the fabrication process and the model in Fig. 2c. However, while the matrix approach provides a powerful tool to analyze the broadband properties of the resonators, a more practical description based on an equivalent circuit model is particularly useful to gain a more informed understanding of the system. For frequencies close to the resonance frequency $f_n$, the impedance of the CPW resonator can be approximated by that of a lumped-elements parallel RLC circuit (Fig. 2d)^28^.

**Figure 3:**
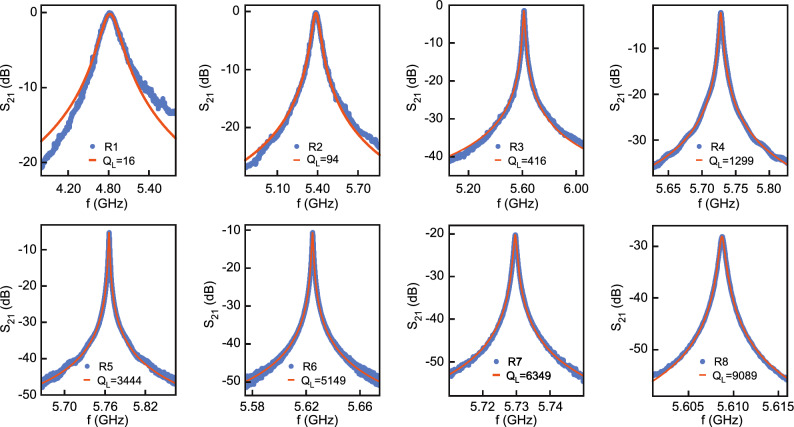
Resonances of the fundamental mode. The figures show the magnitude of $S_{\textrm{21}}$ in dB as a function of frequency at the resonance of the fundamental mode for the eight different resonators at T = 8 K fitted by Eq. (4). The quality factor increases when the coupling capacitance is decreased from left to right.

3 $$\begin{aligned} Z_{\textrm{RLC}}\simeq \frac{R}{1+2iRC(\omega -\omega _n)}, \end{aligned}$$

where $C=C_{\textrm{L}}l/2$, $L=2L_{\textrm{L}}l/\pi ^2$, $\omega _n=n/\sqrt{LC}$ and $R=Z_0/(\alpha l)$. The circuit internal quality factor $Q_{\textrm{int}}=\omega _nRC$ is controlled by the dissipation processes taking place in the resonator, represented by the loss parameter $\alpha$. The transmission coefficient of the resonators can then be written as

4 $$\begin{aligned} S_{\textrm{21}}(\omega )\simeq \frac{\frac{q}{q+1}}{1+2iQ_{\textrm{L}}\left( \frac{\omega }{\omega _n}-1\right) }, \end{aligned}$$

where $Q_{\textrm{L}}$ = $\Big (\frac{1}{Q_{\textrm{int}}}+\frac{1}{Q_{\textrm{c}}}\Big )^{-1}$ is the loaded quality factor of the resonator, expressed as a parallel combination of $Q_{\textrm{int}}$ and $Q_{\textrm{c}}$, and $q=Q_\mathrm{int}/Q_\mathrm{c}$ is the ratio of the two. The square modulus of the transmission coefficient, $|S_\mathrm{21}(\omega )|^2$, takes then a standard Lorentzian form. The coupling quality factor $Q_\mathrm{c}=\omega _nR^*C/2$ quantifies the rate at which the energy stored in the resonator escapes into the external circuit. We have introduced $R^*=\frac{1+\omega _n^2C_c^2R_L^2}{\omega _n^2C_c^2R_L}$, which plays the role of a frequency-dependent effective resistance in the expression of $Q_\mathrm{c}$^25^. Similarly, we also define an effective capacitance $C^*=\frac{C_c}{1+\omega _n^2C_c^2R_L^2}$. $R^*$ and $C^*$ can be considered as the resistance and capacitance of a parallel circuit equivalent to the circuit consisting of $C_c$ in series with $R_L$ (Fig. 2d). Large coupling capacitances slightly decrease the resonance frequency $\omega _n=\frac{n}{\sqrt{L(C+2C^*)}}$. At resonance, the insertion loss, IL, is given by

5 $$\begin{aligned} \mathrm {IL(dB)}=-10\log |S_\mathrm{21}(\omega =\omega _n)|^2=-20\log \left( \frac{q}{q+1}\right) \end{aligned}$$

The *q* coefficient sets the coupling type: for $q\gg 1$, the resonator is in the overcoupled regime with $Q_{\textrm{L}}\approx Q_\mathrm{c}$, and for $q\ll 1$, the resonator is in the undercoupled regime with $Q_{\textrm{L}}\approx Q_\mathrm{int}$. Fig. 2b shows an example of Lorentzian fits performed on the first three resonance modes. The agreement with experimental data is very good close to the resonance frequencies $f_n$.

**Figure 4:**
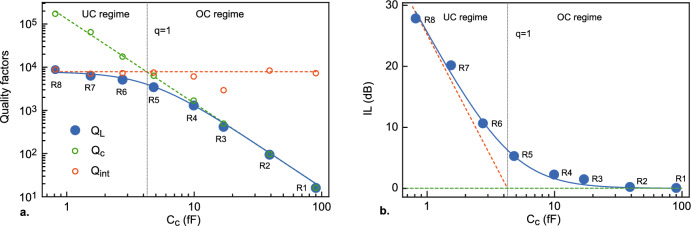
Evolution of quality factors and insertion loss with the coupling. (**a**) Quality factors $Q_{\textrm{L}}$, $Q_\mathrm{int}$ and $Q_\mathrm{c}$ of the resonators measured as a function of the coupling capacitances $C_c$ at T = 8 K. The blue line is a fit to the Lorentzian model. The green and red dashed lines correspond to the limiting cases $Q_\mathrm{c} \simeq C/(2\omega _0R_LC_c^2)$ and $Q_\mathrm{int}=\frac{\pi }{2\alpha l}$ respectively. The critical coupling *q* = 1 separating the undercoupled (UD) and the overcoupled (OC) regimes is reached for $C_c$ $\simeq$ 4 fF. (**b**) Insertion loss as a function the coupling capacitance fitted by the Lorentzian model.

**Figure 5:**
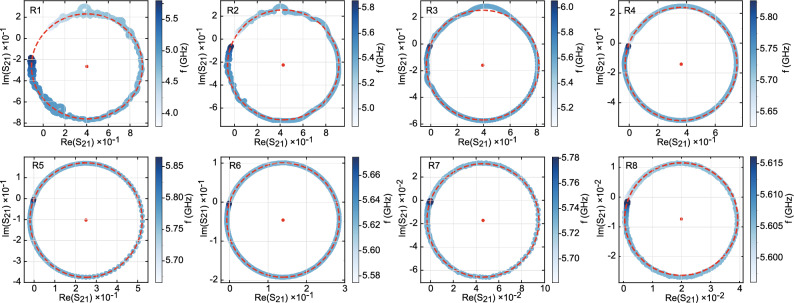
Resonance Circles. Resonant curves of the fundamental mode for the eight resonators showing the real part (bottom axis) and imaginary part (left axis) of $S_\mathrm{21}$ as a function of frequency (color scale) fitted by a circle in the Lorentzian approximation (red dashed line). The diameter of the circles corresponds to the ratio $Q_{\textrm{L}}/Q\textrm{c}$. The red dot indicates the center of the circle.

We now focus on the analysis of the fundamental mode within the Lorentzian approximation. Figure 3 shows the transmission measurements of all resonators close to $f_1$ = $\omega _1/2\pi$, from the highest to the lowest value of the coupling capacitance. Curves are fitted by a Lorentzian function (Eq. 4) of full width at half maximum $\delta _f$ centered on $f_1$, providing the values of $Q_{\textrm{L}}$ and *q*. A very good agreement is observed between the data and the model with the exception of resonator R1, which has a too large bandwidth to really satisfy the Lorentzian approximation. A continuous evolution of the quality factor and insertion loss is observed covering the two different coupling regimes (Fig. 4). In the overcoupled regime ($q\gg 1$), $Q_{\textrm{L}}$ $\simeq$ $Q_\mathrm{c}$ $\simeq$ $C/(2\omega _0R_LC_c^2)$ is entirely determined by $C_c$ (green dashed line in Fig. 4a) and the resonator has a perfect transmission (IL $\simeq$ 0, Fig. 4b). In the undercoupled regime ($q\ll 1$) the resonator is isolated from the external circuit and its quality factor is dominated by the internal losses, $Q_{\textrm{L}}$ $\simeq$ $Q_\mathrm{int}$. This translates into a strong increase of the insertion loss, IL $\simeq$ -20$\log (q)$. In both regimes, the evolution of $Q_{\textrm{L}}$ with $C_c$ is well described within the Lorentzian approximation framework. The overall dependence of our $\textrm{YBa}_2\textrm{Cu}_3\textrm{O}_{7-\delta }$ resonators on the coupling is similar to that observed on conventional low-T$_c$ superconducting resonator^25^ but with reduced internal quality factors. Another way to examine the resonance curves is to plot $S_\mathrm{21}$ in the complex plane as a function of frequency as presented in Fig. 5. In the Lorentzian approximation, the corresponding plot can be fitted by a circle (red dashed lines), whose diameter is defined by the ratio $Q_{\textrm{L}}$/$Q_\mathrm{c}$. While this diameter is close to one in the overcoupled regime (R1, R2, R3 and R4), it strongly decreases in the overcoupled one (R6, R7 and R8) as the losses ($Q_\mathrm{int}$) start to dominate $Q_{\textrm{L}}$. The position of the circle center with respect to the imaginary axis shifts from $Im(S_\mathrm{21})$ < 0 for large value of the coupling capacitors $C_c$ towards $Im(S_\mathrm{21})$ = 0 for very small capacitors, which corresponds to the expected limit for the pure Lorentzian form.

**Figure 6:**
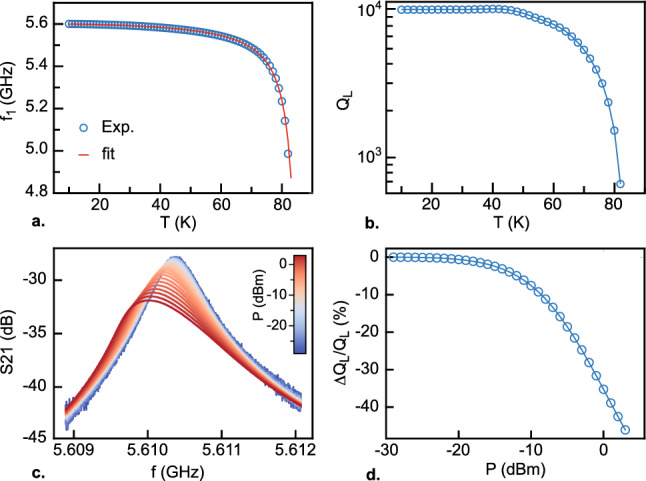
Evolution of resonator R8 with temperature and microwave power. (**a**) Resonance frequency as a function of temperature (open symbols) fitted by the Gorter–Casimir model (Eq. 6). (**b**) Loaded Quality factor as a function of temperature. c) Resonance curves for different microwave powers at the input of the resonator (color scale). (**c**) Relative variation of the quality factor of $Q_{\textrm{L}}$ as a function of input power extracted from panel c.

The properties of resonator R8 were further studied as a function of temperature and microwave power. Figure 6a shows the temperature dependence of its fundamental resonance frequency $f_1=\frac{1}{2\pi \sqrt{LC}}$, where *C* and *L* are the effective capacitance and inductance introduced in the Lorentzian approximation (Eq. 3). While the kinetic inductance played a negligible role in the low-temperature range discussed earlier, it must be taken into account for analyzing the complete temperature dependence. The total inductance *L* is therefore the sum of the geometric inductance $L_\mathrm{G}=2L_{\textrm{L}}l/\pi ^2$ and the kinetic inductance of the superconductor $L_k(T)$ that varies with temperature. At low temperatures, *L* is predominantly determined by $L_\mathrm{G}$, leading to the saturation of $f_1$ below 15 K. However, at the critical temperature $T_c$, $L_k$ diverges, resulting in a pronounced shift of $f_1$ towards lower frequencies. In the Gorter–Casimir two fluid model, the temperature dependent kinetic inductance of a superconductor is given by^31^

6 $$\begin{aligned} L_k(T)=\frac{L_0}{1-\left( \frac{T}{T_c}\right) ^a} \end{aligned}$$

where $L_0$ is the zero temperature value of the inductance that dependents on the exact geometry of the superconductor and its London penetration length $\lambda _0$. The best fit yields an exponent *a* $\simeq$ 2.09, which aligns well with values close to 2 typically observed for high-quality high-T$\textrm{c}$ superconductors.^27,32–34^.

We now discuss the temperature dependence of the quality factor of resonator R8 shown in Fig. 6b. As seen previously, this resonator is deeply in the undercoupled regime for which $Q_{\textrm{L}}$ $\approx$ $Q_\mathrm{int}$. In planar resonators, different mechanisms can contribute to the losses, which in turn, set the value of the internal quality factor. These include losses in the conducting material, dielectric losses, two-level systems in the substrate, and radiation losses. However, in resonators fabricated using high-T$_c$ superconducting materials, conductor losses represent the dominant contribution to the overall losses in the system. As depicted in Fig. 6b, the quality factor rises when the temperature is decreased below T$_c$. This improvement can be attributed to the reduction in the number of quasiparticles in $\textrm{YBa}_2\textrm{Cu}_3\textrm{O}_{7-\delta }$ as the superconducting gap becomes larger. At temperatures below 40 K, the quality factor reaches a saturation point, mainly due to residual losses. Additional measurements in a dilution refrigerator show that the value of the quality factor remains constant down to 100 mK (Supplementary Fig. 2). In high-T$_c$ superconducting thin films, residual losses are commonly attributed to weak-links caused by grain boundaries. Additionally, vortices can also contribute to residual losses as confirmed by the variation of the quality factor in the presence of an external magnetic field (see Supplementary Information)^35^. In this work, we obtained a maximum quality factor $Q_\mathrm{int} \simeq 9100$ for resonator R8 corresponding to an attenuation constant $\alpha$ $\simeq$ 1.5 $\times$10$^{-2}$ m$^{-1}$. The microwave losses in thin films are often described in terms of surface resistance which can be estimated from the value of $\alpha$^34^. Assuming a typical value of the London penetration depth $\lambda _0$ $\simeq$ 180 nm, we obtain a surface resistance $R_s$ $\approx$ 40 $\mu \Omega$ at T = 8 K, which is in good agreement with values reported in the literature for resonators in the same frequency range^35,36^. The value of $\alpha$, extracted from the resonance of the harmonic modes at higher frequencies, indicates that conductor losses tend to increase linearly with frequency (Supplementary Fig. 3).

The presence of grain boundaries and vortices in the $\textrm{YBa}_2\textrm{Cu}_3\textrm{O}_{7-\delta }$ thin films is a natural source of non-linearity at high microwave power. Figure 6c illustrates the evolution of the resonance curves of resonator R8 when subjected to high microwave power at its input. Beyond *P* = −15 dBm, the resonance starts to exhibit skewing, a characteristic feature of nonlinear oscillators. Additionally, $f_1$ decreases, and the bandwidth undergoes a significant increase. However, as observed in Fig. 6d, which represents the relative variation of the quality factor as a function of input power, $Q_{\textrm{L}}$ remains constant over a wide power range. It begins to decrease significantly only for relatively high powers, around *P* = −15 dBm, corresponding to a substantial number of photons in the cavity, approximately ${\bar{n}}$ $\simeq$ 6 $\times$ 10$^{11}$. The sources of dissipation and non-linearity in high-T$_c$ superconducting resonators are mainly extrinsic and can vary significantly depending on the quality of the films and in particular the specific growth method. Higher quality factors could be reached by optimizing the resonator geometry such as using a thicker $\textrm{YBa}_2\textrm{Cu}_3\textrm{O}_{7-\delta }$ film and a wider central conductor, while maintaining its characteristic impedance constant. The impact of vortices can be mitigated by employing $\textrm{YBa}_2\textrm{Cu}_3\textrm{O}_{7-\delta }$ superconducting films with increased surface roughness, leading to enhanced vortex pinning. Quality factors larger than 250,000 were already reported in very large and lower frequency $\textrm{YBa}_2\textrm{Cu}_3\textrm{O}_{7-\delta }$ LC resonators^37^.

## Electron spin resonance

In the following, we examine the potential of our resonators to perform Electron Spin Resonance (ESR) experiments on a spin ensemble. The high-Q resonator R8 was connected to a microwave printed circuit board and a 0.25 mg pellet of DPPH powder containing $N_0$ $\approx$ 3.8 $\times$ 10$^{17}$ spins was deposited at the center of the resonator. The DPPH molecule possesses a highly stable free radical whose orbital motion is quenched and behaves like a spin 1/2, which is commonly used as a reference marker in ESR spectroscopy. The sample was cooled down in a dilution refrigerator equipped with a superconducting magnet producing an in-plane Zeeman magnetic field *B*. The microwave signal was coupled to the resonator through a 45 dB attenuated line and the transmitted signal was amplified at the 3 K stage by a low noise cryogenic HEMT amplifier. Two isolators at the 100 mK and 3 K stages were included between the sample and the amplifier to limit the back action noise on the device. All the experiments were performed with an average number of photons ${\bar{n}}$ $\approx$ 1.3 $\times$ 10$^9$ in the cavity, which is deduced from the input power and quality factor^38^. The quality factor of the resonator was only marginally affected by the magnetic field used in this study (B $\simeq$ 200 mT) and a decrease of only 35$\%$ in the quality factor value was observed at 6 T (Supplementary Fig. 1), demonstrating that $\textrm{YBa}_2\textrm{Cu}_3\textrm{O}_{7-\delta }$ resonators can operate under large magnetic fields in agreement with previous work^16^.

**Figure 7:**
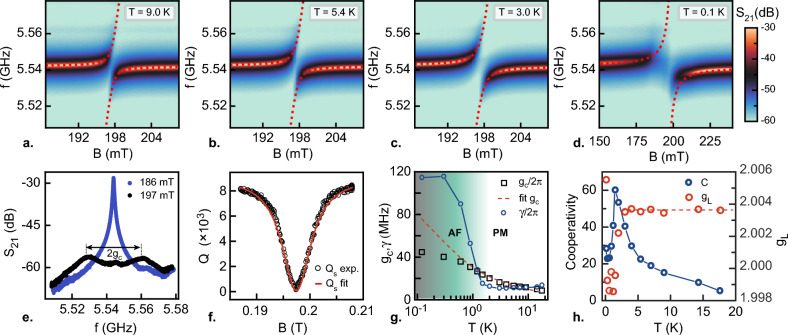
ESR experiment on DPPH. (**a**–**d**) Magnitude of $S_\mathrm{21}$ in color scale as a function of frequency and magnetic field measured on resonator R8 in presence of DPPH for four different temperatures. The dashed line on each panel corresponds to a fit of the Rabi spliting (Eq. 8). (**e**) ESR spectrum for two values of the magnetic field *B* measured at T = 3 K. At the spin resonance $B_0$ = 197 mT, two peaks separated by $2g_c$ are visible corresponding to the two branches of the Rabi splitting. (**f**) Quality factor of the resonance as a function of magnetic field fitted to Eq. (9). (**g**) Coupling ($g_c$) and spin decay rate ($\gamma$) as a function of temperature extracted from the fitting procedure. The red dashed line is a fit to the expression $g_c=g_s\sqrt{N_0\tanh \bigg (\frac{hf_1}{2k_BT}\bigg )}$. For T > 2 K, the spins are in a paramagnetic (PM) state and for T < 2 K antiferromagnetic (AF) correlations generate an increase of $\gamma _s$. (**h**) Cooperativity (left axis) and Landé g-factor (right axis) and as a function of temperature. The dashed line corresponds to $g_{\textrm{L}}$ $\simeq$ 2.0037 which has been used to calibrated our magnetic field at *T* > 2 K.

Figure 7 shows examples of the transmission spectra in color scale as a function of the Zeeman magnetic field *B* at four different temperatures. The spin resonance occurs for $\hbar \omega _1$ = $g_{\textrm{L}}\mu _B B_0$^39^ corresponding to a magnetic field $B_0$ $\simeq$ 197 mT ($g_{\textrm{L}}$ $\simeq$ 2.0037 is the Landé g-factor of DPPH). In addition to the strong suppression of the transmission peak, an avoided crossing is observed, revealing a spin-cavity hybridization as expected in the framework of the Jaynes–Cummings model which describes the interaction between a quantized electromagnetic field and a two-level quantum system^40^. At the spin resonance, the eigenstates of the system are a balanced superposition of mixed spin-photon states of the form ${|{\Psi _\pm }\rangle }=\frac{1}{\sqrt{2}}\big ({|{{\uparrow ,0}}\rangle }\pm {|{\downarrow ,1}\rangle }\big )$. While the magnetic dipole coupling of a single spin to the electromagnetic field, $g_s$, is in principle very weak, an ensemble of independent spins generates an enhanced effective collective coupling $g_c$. For a small excitation, corresponding to a number of photons in the cavity much lower than the number of polarized spins, $g_c=g_s\sqrt{N(T)}$, where *N*(*T*) is the temperature-dependent number of polarized spins^41^. In presence of the spin ensemble, the transmission of the resonator becomes^42^

7 $$\begin{aligned} S^s_{21}(\omega ,\omega _L)\simeq \frac{\omega _1/Q_\mathrm{c}}{\omega _1/Q+2i(\omega _1-\omega )+\frac{2g_c^2}{i(\omega _L-\omega )+\gamma _s}}, \end{aligned}$$

where $\omega _L$ = $g\mu _BB/\hbar$ is the Larmor frequency and $\gamma _s$ is the decay rate of the spin ensemble. Close to the spin resonance, $|S^s_{21}(\omega ,\omega _L)|^2$ displays two resonance frequencies that correspond to the eigenenergies of the hybridized states (neglecting the damping $\gamma _s$)

8 $$\begin{aligned} \omega _{\pm }=\omega _1+\frac{\Delta }{2}\pm \frac{\sqrt{\Delta ^2+4g^2_c}}{2}, \end{aligned}$$

where $\Delta$ = $g\mu _B(B-B_0)/\hbar$ is the detuning. At the spin resonance, the two Rabi branches are exactly separated by 2$g_c$ (Fig. 7e). For each branch ($\omega _{+}$, $\omega _{-}$) , $|S^s_{21}(\omega )|^2$ can be locally approximated by a Lorentzian form with a quality factor^18^

9 $$\begin{aligned} Q_\mathrm{s}=\frac{\Delta ^2+\gamma _s^2}{2g_c^2\gamma _s+\kappa (\Delta ^2+\gamma _s^2)}\omega _1, \end{aligned}$$

This expression is valid for the range of parameters covered in this work. While for large detunings $Q_\mathrm{s}\simeq \frac{\omega _1}{\kappa }=Q_{\textrm{L}}$, the decrease of $Q_\mathrm{s}$ when approaching the resonance, is controlled by the spin-photon coupling and the spin decay rate ($Q_\mathrm{s}\simeq \frac{\gamma _s\omega _1}{4g_c^2}$).

At each temperature, the transmission of the resonator as a function of frequency and magnetic field, was fitted to Eq. (7), which provides the values of the parameters $g_c$ and $\gamma _s$. The frequencies $\omega _{\pm }$ and quality factor $Q_\mathrm{s}$ are then calculated with Eqs. (8) and (9) respectively. In both cases, a good agreement is found with the experimental data, as seen in Fig. 7 (panels a,b,c and d for $\omega _{\pm }$ and panel f for $Q_\mathrm{s}$). The temperature dependence of the effective coupling constant extracted from the fitting procedure is shown in Fig. 7g. The coupling $g_c$ varies from 8.6 MHz at T = 17.4 K to 35 MHz at T = 0.1 K. We first focus on temperatures above 2 K corresponding to data points on the right region in Fig. 7g. In this range, the temperature dependence of $g_c(T)$ is consistent with a thermal depolarization of the spin ensemble described by a Boltzmann statistics $g_c=g_s\sqrt{N_0\tanh \bigg (\frac{hf_1}{2k_BT}\bigg )}$. Given the estimated total number of spins $N_0$ $\approx$ 3.8 $\times$ 10$^{17}$ contained in the DPPH pellet, we deduce a single spin coupling constant $g_s$ $\simeq$ 0.15 Hz, consistent with the geometry of the resonator and previous studies^16^. In the same temperature range, $\gamma _s$ slightly decreases when lowering the temperature, which could be a consequence of exchange narrowing at low temperature^43^. The spin decay rate $\gamma _s$ reaches a minimum value of 11 MHz at 2 K corresponding to a relaxation time of 90 ns. The coupling efficiency between the cavity and the spin can be evaluated through the dimensionless cooperativity parameter $C_\mathrm{op}=g_c^2/(\kappa \gamma _s)$ (Fig. 7h). Throughout the whole temperature range, the system is in a highly cooperative regime ($C_\mathrm{op}$ $\gg$ 1), indicating coherent transfer of photons between the electromagnetic field and the spin ensemble before spin relaxation. The strong coupling regime conditions, $g_c\gg \kappa ,\gamma _s$, for which the coupling is faster than the decay rate of both the cavity and the spin ensemble are also partially satisfied.

For T < 2 K, a significant increase of the spin linewidth $\gamma _s$ is observed, indicating the appearance of correlations between spins. Likewise, the temperature dependence of the coupling $g_c$ starts to deviate from its expected trend, indicating that the spins are no longer independent and therefore do not provide the $\sqrt{N(T)}$ enhancement of the collective coupling to the cavity (Fig. 7g). These observations are also reinforced by the drop of the Landé g-factor extracted experimentally from the value of the magnetic field at the resonance $B_0$ and the drop of the cooperativity below 2 K (Fig. 7h). We ascribe these phenomena to an emerging antiferromagnetic state, previously reported in DPPH at a Néel temperature $T_N$ $\approx$ 0.4 K in monocrystals^44,45^. However, in crystalline powder, signatures of the transition have been identified at higher temperatures, up to 2 K, in agreement with our observation and possibly related to a stronger exchange interaction^44^. A similar increase in spin linewidth has also been observed recently in van der Waals compounds when approaching an antiferromagnetic transition^46^. Our ESR technic is therefore particularly interesting to probe the spin dynamics in insulating magnetic materials.

## Conclusion

In summary, we have fabricated $\textrm{YBa}_2\textrm{Cu}_3\textrm{O}_{7-\delta }$ CPW superconducting resonators and evidenced two different operating regimes depending on the geometry : an overcoupled lossless regime, where the quality factor is controlled by the coupling to the external circuit, and an undercoupled dissipative one, where the quality factor is limited by the intrinsic losses in the resonator. While a maximum value of $Q_{\textrm{L}}\approx 9100$ has been demonstrated in this geometry, higher values of the quality factor could be reached by optimizing the resonator design. In addition, we have successfully used a high-Q resonator to perform an ESR experiment on DPPH crystalline powder containing $N_0$ $\approx$ 3.8 $\times$ 10$^{17}$ molecular spins, even though the resonator geometry was not optimized for high spin sensitivity. The ESR spectra reveal a spin-cavity hybridization in the strong cooperative regime with a double peak structure due to a Rabi splitting. The analysis of the spin-cavity coupling strength, the spin decay rate, and the Landé g-factor point toward an antiferromagnetic transition at low temperatures. Our findings demonstrate that $\textrm{YBa}_2\textrm{Cu}_3\textrm{O}_{7-\delta }$ resonators hold promising potential for realizing hybrid quantum systems, where quantum information could be stored or processed. However, despite achieving the strong coupling regime in our ESR experiment, both the spin and photon lifetimes within the cavity fall short for practical quantum memory applications. To overcome this technological limitation, resonators with a quality factor larger than 50000 are needed, which requires further development. In a broader scope, $\textrm{YBa}_2\textrm{Cu}_3\textrm{O}_{7-\delta }$ resonators can be valuable in applications requiring high magnetic fields (up to a few T) and operating at intermediate cryogenic temperatures (below 50 K). Some of these applications include, but are not limited to, ESR experiments in high magnetic fields and the study of excitations in magnetic materials.

### Supplementary Information

Supplementary Information.

## Data Availability

The data used in this study are available from the corresponding author on reasonable request.
