# Author Correction: Hybrid quantum systems with high-T_*c*_ superconducting resonators

**DOI:** 10.1038/s41598-023-44720-4

**Published:** 2023-10-13

**Authors:** Z. Velluire-Pellat, E. Maréchal, N. Moulonguet, G. Saïz, G. C. Ménard, S. Kozlov, F. Couëdo, P. Amari, C. Medous, J. Paris, R. Hostein, J. Lesueur, C. Feuillet-Palma, N. Bergeal

**Affiliations:** 1 Laboratoire de Physique et d’Étude des Matériaux, ESPCI Paris, Université PSL, CNRS, Sorbonne Université, Paris, France; 2 Laboratoire National de Métrologie et d’Essais (LNE), 29 Avenue Roger Hennequin, 78197 Trappes, France; 3 CNRS, Institut Fourier, Université Grenoble Alpes, 38000 Grenoble, France; 4 Université Grenoble Alpes, INRIA, 38000 Grenoble, France; 5 My Cryo Firm, 20 Villa des Carrières, 94120 Fontenay-sous-Bois, France

Correction to: *Scientific Reports* 10.1038/s41598-023-41472-z, published online 01 September 2023

The original version of this article contained an error in the spelling of the author Z. Velluire-Pellat which was incorrectly given as Z. Velluire Pellat.

The original article has been corrected.

