# Supplementary material for: Hybrid quantum systems with high-Tc superconducting resonators
Source: Sci Rep. 2023 Sep 1;13:14366. doi: 10.1038/s41598-023-41472-z (PMC10474070; doi:10.1038/s41598-023-41472-z)
Supplement: Supplementary file 1 — Supplementary Information. [file 41598_2023_41472_MOESM1_ESM.pdf]

Supplementary Information : Hybrid Quantum Systems with High-T$_c$ Superconducting Resonators

Z. Velluire Pellat, E. Maréchal, N. Moulonguet, G. Saïz, G. C. Ménard, S. Kozlov, F. Couëdo, P. Amari, C. Medous, J. Paris, R. Hostein, J. Lesueur, C. Feuillet-Palma, N. Bergeal

I. Behavior of resonator R8 under a magnetic field

In Supplementary Figure 1, we show the dependence of the resonance frequency and quality factor of resonator R8 as a function of a magnetic field applied in the resonator plane. For small magnetic fields ($B <$ 0.15 T), we observe a slight increase in both f_1 and Q_L. This phenomenon has been previously reported in superconducting resonators (for instance in Nb), and is often attributed to the spatial redistribution of Abrikosov vortices under a perpendicular magnetic field [1]. Typically, Nb resonators show a maximum quality factor at a few mT when subjected to such fields. Although we apply the magnetic field in plane in our experiment, the alignment between the sample holder plane and the field has a precision of 1-2 degrees, leading to a perpendicular field of a few mT for an applied field $B =$ 150 mT, which is sufficient to generate vortices. The resonator losses due to the presence of the magnetic field are therefore primarily determined by the component of B perpendicular to the sample. For higher fields ($B >$ 0.15 T), we observe a relatively slow decrease in quality factor (Q_L) due to the proliferation of vortices. At 6 T, Q_L is reduced by approximately 35%. However, it's worth noting that this reduction could potentially be optimized with improved sample alignment into the magnetic field. Nonetheless, our experiment demonstrates that $\mathrm{YBa_2Cu_3O_{7-\delta}}$ res-

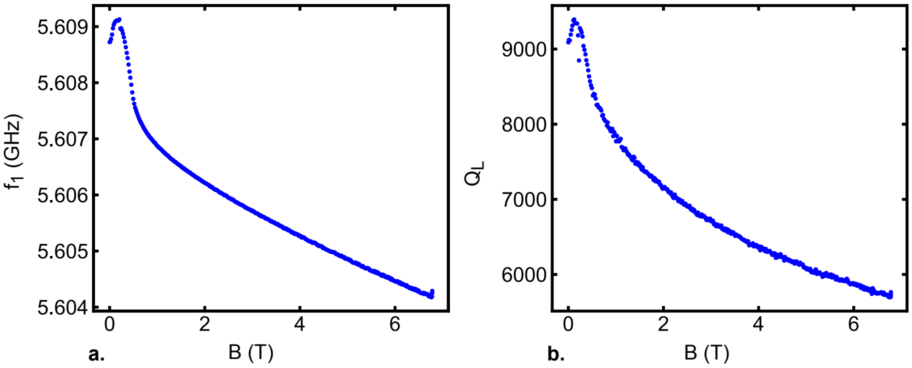

Supplementary Figure 1: a) Resonance frequency of resonator R8 as a function of an in-plane magnetic field B. b) Loaded quality factor of resonator R8 as a function of an in-plane magnetic field B.

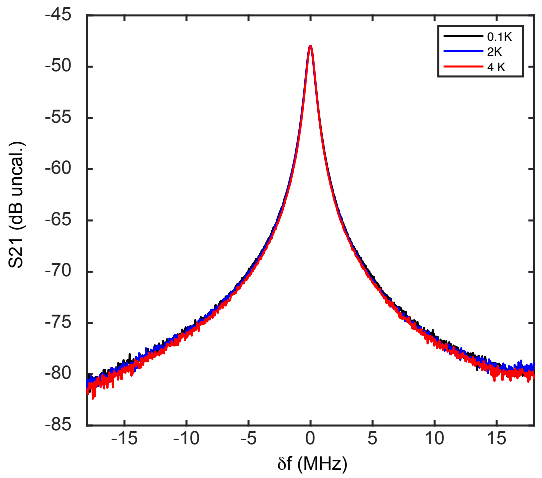

Supplementary Figure 2: Resonance curves of resonator R8 measured in a dilution refrigerator at different temperatures (0.1, 2K and 4K). The quality factor remains constant in the low temperature regime.

onators can operate effectively under strong magnetic fields with only limited effects on the quality factor.

II. Evolution of losses at very low temperature

Figure 6 in the main text shows the evolution of the resonance frequency and quality factor of resonator R8 as a function of temperature measured in the microwave probe station down to 8K. For the purpose of the ESR experiment, the device was later connected to a PCB and measured in a dilution refrigerator having a base temperature of 100mK. As shown in the Supplementary Figure 2 the quality factor only marginally evolves between 100mK and 4K, which rules out a significant effect of two-level systems on the resonator losses [2].

III. Evolution of losses as a function of frequency

In Supplementary Figure 3a, we show the quality factors $Q_{\rm L}$, $Q_{\rm int}$ and $Q_{\rm c}$ extracted from the measurement of resonator R7 for the modes n=1, n=2 and n=3 corresponding to the frequency range accessible in our set-up (0-20GHz). The corresponding attenuation constant α extracted from $Q_{\rm int}$ of the higher frequency harmonics indicates that conductor losses tend to increase linearly with frequency. This increase is stronger than the expected linear increase of $Q_{\rm int} = \omega_n RC = n\pi/(2\alpha l)$ with the mode number (i.e. with the frequency) which results in a limited reduction of $Q_{\rm int}$ from 7000 at 5.5GHz to 4000 at 16.5 GHz approximately.

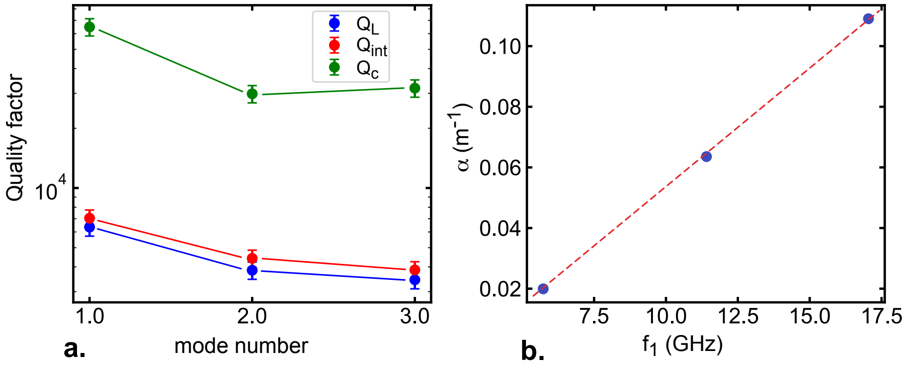

Supplementary Figure 3: a) Loaded (Q_L), internal (Q_int) and Coupling (Q_c) quality factors of resonator R7 as a function of the mode number n =1, 2 and 3. b) Evolution of the attenuation constant α as a function of frequency extracted from Q_int for the different modes.

[1] Bothner, D., Gaber, T., Kemmler, M., Koelle, D., Kleiner, R., Wünsch, S. and Siegel, M. Magnetic hysteresis effects in superconducting coplanar microwave resonators. *Phys. Rev. B* **86**, 014517 (2012).

[2] Arzeo, M., Lombardi, F. and Bauch. T. Microwave Losses in YBCO Coplanar Waveguide Resonators at Low Power and Millikelvin Range, *IEEE Trans. Appl. Supercond.* 3, 25, (2015).
